# Developmental ecology of the infant oral ecosystem: a framework for early childhood caries

**DOI:** 10.3389/froh.2026.1956432

**Published:** 2026-09-04

**Authors:** Mariarosaria Matera, Alberto Besostri, Ilaria Cavecchia, Maria Teresa Illiceto, Maria Beatrice Lenzi, Talia D’Ambrosio, Maria Elisabetta Baldassarre, Valentina Biagioli

**Affiliations:** 1 Microbiota International Clinical Society, Turin, Italy; 2 Department of Pediatric Emergencies, Misericordia Hospital, Grosseto, Italy; 3 Preventive and Community Dentistry, School of Dental Hygiene, University of Padova, Padova, Italy; 4 Microbiomic Department, Kooelliker Hospital, Turin, Italy; 5 Unit of Pediatric Gastroenterology and Digestive Endoscopy, Department of Pediatrics, Santo Spirito Hospital, Pescara, Italy; 6 Department of Neonatology and Neonatal Intensive Care Unit (NICU), Villa Sofia-Cervello Hospital, Palermo, Italy; 7 Department of Pediatric, University of Ferrara, Ferrara, Italy; 8 Primary Care Pediatrician, Azienda Unità Sanitaria Locale Ferrara, Ferrara, Italy; 9 Neonatology and Neonatal Intensive Care Unit (NICU) Section, Department of Interdisciplinary Medicine, University Aldo Moro, Bari, Italy; 10 Department of Neurosciences, Rehabilitation, Ophtalmology, Genetics, Maternal and Child Health, University of Genoa, Genoa, Italy

**Keywords:** developmental ecology, developmental origins of health and disease (DOHaD), early childhood caries, one health, oral ecosystem resilience, oral microbiome

## Abstract

**Background:**

Early childhood caries (ECC) has traditionally been regarded as a biofilm-mediated disease driven by sugar exposure and acidogenic microorganisms. However, this perspective primarily captures the final stage of a much longer biological process. We propose the Developmental Ecology of the Infant Oral Ecosystem as a conceptual framework in which ECC represents the clinical manifestation of a disrupted developmental ecology of the infant oral ecosystem, resulting from altered host-microbe-environment interactions that operate from the prenatal period through the first 1,000 days of life with maternal, nutritional, inflammatory, immune, metabolic, microbial, and environmental influences shaping early developmental trajectories.

**Methods:**

This narrative review synthesizes current evidence into an integrated developmental ecological framework encompassing maternal microbial transmission, infant oral microbiome assembly, breastfeeding, complementary feeding, dietary ecology, tooth eruption, biofilm maturation, and the oral–gut axis. These processes are interpreted within the One Health and Developmental Origins of Health and Disease (DOHaD) paradigms to provide a comprehensive ecological perspective on ECC development.

**Findings:**

The reviewed evidence supports a developmental-ecological interpretation in which interconnected early-life ecological processes shape microbial succession, oral ecosystem resilience, and susceptibility to dysbiosis. Rather than acting as the primary cause of disease, the cariogenic biofilm is interpreted within the proposed framework as the ecological endpoint of disrupted developmental trajectories that impair the establishment and maintenance of oral homeostasis.

**Conclusions:**

Reframing ECC as a developmental ecological disorder complements established preventive approaches, including fluoride use, dietary sugar control, oral hygiene, and regular dental care, while highlighting the importance of maternal health, early-life nutritional ecology, and microbiome-informed preventive strategies aimed at preserving oral ecosystem resilience from the earliest stages of life. The proposed framework is intended to generate testable hypotheses for future longitudinal and interventional research.

## 1 Introduction

Early childhood caries (ECC) remains one of the most prevalent chronic diseases of childhood and continues to represent a major public health challenge despite being largely preventable. Recent systematic reviews estimate that ECC affects approximately 46%–48% of preschool-aged children globally, while the Global Burden of Disease Study indicates that more than 500 million children are affected by caries in primary teeth worldwide (1). The burden of disease is unevenly distributed, disproportionately affecting socioeconomically disadvantaged populations and low- and middle-income countries, where access to preventive oral healthcare remains limited.

Beyond its oral manifestations, ECC is increasingly recognized as a condition with broader implications for child health and well-being. Untreated caries may cause chronic pain, feeding difficulties, impaired mastication, sleep disturbances, reduced quality of life, and increased healthcare utilization. Severe forms of ECC have also been associated with impaired growth, nutritional deficiencies, school absenteeism, and substantial psychosocial and economic consequences for affected children and their families (2, 3). Emerging evidence further suggests that severe untreated ECC may reflect broader biological, nutritional, and environmental vulnerabilities operating during early life, reinforcing the need for preventive strategies that extend beyond oral health alone (4, 5).

Historically, dental caries was regarded as an infectious disease caused primarily by specific pathogenic microorganisms, particularly *Streptococcus mutans*. This concept was strongly influenced by the Specific Plaque Hypothesis proposed by Loesche, which postulated that only a limited number of bacterial species within dental plaque were directly responsible for disease development (6). However, advances in microbial ecology and microbiome science have progressively challenged this reductionist view, revealing that oral health reflects dynamic interactions among microbial communities, host biological factors, diet, and environmental influences (7, 8). Host-related determinants, including enamel development, salivary function, immune responses, and genetic susceptibility, may further modulate the risk of ecological dysbiosis and disease (9, 10).

This conceptual evolution culminated in the Ecological Plaque Hypothesis proposed by Marsh, which posits that environmental changes within the biofilm drive microbial selection and ultimately determine disease onset (11). According to this model, repeated exposure to fermentable carbohydrates and acidification promotes the selection of acidogenic and aciduric microbial communities, leading to dysbiosis, reduced microbial diversity, and loss of ecosystem resilience (12, 13).

Consequently, dental caries is increasingly viewed as a microbiome-mediated ecological disease resulting from loss of ecosystem resilience rather than infection by a single pathogen. While the ecological plaque hypothesis has fundamentally transformed our understanding of caries pathogenesis, increasing evidence suggests that the foundations of oral health are established much earlier in life.

Building upon this ecological perspective, we propose the Developmental Ecology of the Infant Oral Ecosystem as a conceptual framework in which Early Childhood Caries represents the clinical manifestation of the disrupted developmental ecology of the infant oral ecosystem, operating from pregnancy through the first 1,000 days of life. Within this framework, the cariogenic biofilm represents the ecological endpoint rather than the primary driver of disease.

Maternal microbiota, mode of delivery, breastfeeding, family microbial sharing, dietary exposures, and environmental factors collectively shape the infant oral ecosystem and may influence long-term oral health trajectories (14, 15).

From this perspective, oral health can be viewed through a One Health lens, recognizing that microbial communities develop within interconnected biological, behavioral, and environmental systems (5). The infant oral microbiome emerges not in isolation but within a shared Mother-Infant-Environment ecosystem, where maternal microbial transmission, family interactions, dietary exposures, and environmental influences collectively shape microbial succession (14, 16). Early-life exposures occurring during critical developmental windows may influence microbial succession, ecosystem resilience, and susceptibility to dysbiosis, thereby shaping long-term oral health trajectories (8, 17). Understanding these interactions may provide new opportunities for preventive strategies aimed at preserving oral ecosystem health from the earliest stages of life.

This narrative review aims to provide an integrated ecological framework linking maternal oral microbiota, early microbial transmission, infant oral ecosystem development, dietary ecology, and biofilm maturation in ECC pathogenesis. By adopting a Mother-Infant-Environment perspective grounded in One Health principles, this review integrates current evidence into a developmental ecological framework that explains how early-life ecological processes shape oral ecosystem resilience and influence the emergence of the cariogenic biofilm.

## 2 Materials and methods

### 2.1 Study design

This study was designed as a narrative review aimed at developing an integrated conceptual framework describing the developmental ecology of the infant oral ecosystem and its role in the pathogenesis of early childhood caries (ECC). Rather than following a systematic review methodology, evidence from oral microbiology, microbiome science, developmental biology, nutrition, pediatrics, ecology, and One Health was critically integrated to develop the proposed framework.

### 2.2 Literature search strategy

A literature search was conducted in the PubMed/MEDLINE database and included articles available through June 2026. No lower date limit was applied to include both landmark studies that established the conceptual foundations of dental caries and oral microbial ecology and contemporary evidence on early-life determinants of oral health.

Searches included combinations of the following terms: early childhood caries, oral microbiome, Ecological Plaque Hypothesis, maternal oral microbiota, maternal–infant microbial transmission, breastfeeding, human milk, first 1,000 days, complementary feeding, biofilm, microbial succession, dysbiosis, and One Health. Because the objective was to develop a conceptual framework, no formal systematic review protocol was followed.

### 2.3 Evidence selection and conceptual synthesis

Articles were selected according to their relevance to the objectives of the review and their contribution to understanding the biological, ecological, developmental, and environmental determinants of ECC. Priority was given to systematic reviews, meta-analyses, longitudinal cohort studies, prospective clinical investigations, mechanistic studies, and international consensus documents. Seminal publications were retained when essential to contextualize the evolution of oral microbial ecology and dental caries research.

The selected evidence was critically integrated to reconstruct the evolution of scientific concepts underlying ECC pathogenesis. This conceptual synthesis culminated in the Developmental Ecology of the Infant Oral Ecosystem framework proposed in this review, which integrates contemporary evidence from microbiome science, developmental biology, nutritional ecology, and early-life determinants into a unified ecological perspective.

## 3 Building the infant oral ecosystem: the mother-infant-family axis

### 3.1 Maternal oral microbiota and early oral microbial transmission

The maternal oral microbiota is one of the earliest microbial reservoirs shaping the infant oral ecosystem. Although the infant's oral cavity is rapidly exposed to microorganisms from multiple sources after birth, maternal microbial communities play a pivotal role in initiating colonization through the first ecological inoculum (11, 14). The composition of the maternal oral microbiome influences the pool of microorganisms available for transmission, thereby shaping early microbial assembly in the offspring (18).

Pregnancy is associated with significant physiological, hormonal, and immunological changes that can alter the composition and diversity of the oral microbiota (19). Increased levels of estrogen and progesterone, together with pregnancy-associated immune modulation, may favor shifts in microbial composition and increase susceptibility to oral conditions such as gingivitis and periodontal inflammation (20). Consequently, maternal oral health directly influences the microbial reservoir available for early transmission to the infant (21). Poor oral health and periodontal disease have been associated with increased abundance of pathogenic taxa, which may influence early microbial exposure and colonization patterns (21).

Several maternal factors further contribute to shaping the oral microbiome during pregnancy. Environmental exposures, dietary habits, smoking, oral hygiene practices, and antibiotic use can modify microbial composition and affect microbial diversity (22–24). Antibiotic exposure, in particular, may disrupt microbial homeostasis, while dietary patterns influence the availability of substrates that support specific microbial populations (25). Collectively, these factors generate interindividual variability in maternal microbial profiles, influencing the ecological inheritance transmitted to the offspring.

Early microbial acquisition occurs through both vertical and horizontal transmission pathways. Vertical transmission primarily involves direct transfer of maternal microorganisms through close contact, saliva exchange, and caregiving behaviors (26). However, the infant oral microbiome is also shaped by horizontal transmission from fathers, siblings, caregivers, and the surrounding environment. Increasing evidence suggests that family members share microbial taxa, generating a household-specific microbial signature that contributes to the development of the infant microbiome (18). These early transmission events provide the initial microbial inoculum upon which ecological succession progressively unfolds throughout infancy. Importantly, maternal microbial transmission may extend beyond direct oral colonization. Swallowed oral microorganisms can reach the gastrointestinal tract, and under conditions of oral dysbiosis, oral pathobionts may transiently colonize the infant gut and potentially influence mucosal immune development (27, 28).

The assembly of the infant oral microbiota is a dynamic ecological process characterized by sequential colonization events. Pioneer species establish shortly after birth and modify the local environment, facilitating the recruitment of additional microorganisms (14). As the infant grows, ecological succession drives progressive increases in microbial diversity and community complexity. Major developmental milestones, including tooth eruption, dietary diversification, and maturation of host immune functions, create new ecological niches that further shape microbial composition (29, 30). Overall, the infant oral microbiome emerges through continuous ecological inheritance driven by maternal, familial, host, and environmental interactions rather than isolated transmission events. This developmental continuity provides the biological foundation for subsequent dietary, behavioral, and host influences that shape oral ecosystem maturation throughout early childhood.

### 3.2 Breastfeeding as a biological bridge between maternal and infant ecosystems

Breastfeeding represents a unique biological interface through which maternal signals are continuously transmitted to the infant, shaping microbial colonization, immune maturation, and developmental programming. Beyond providing nutrients, human milk acts as a complex bioactive system that connects maternal and infant ecosystems through the transfer of microorganisms, immune factors, metabolites, and signaling molecules (31).

The milk microbiota contributes to the establishment of the neonatal gut microbiome by delivering commensal bacteria, including *Bifidobacterium*, *Lactobacillus*, and *Streptococcus* species. This process is further supported by human milk oligosaccharides (HMOs), complex glycans that selectively promote the growth of beneficial microbes while limiting pathogen colonization. Through their prebiotic and immunomodulatory properties, HMOs contribute to the development of a balanced microbial ecosystem and mucosal homeostasis (32–34).

Beyond its microbial component, human milk provides a broad repertoire of immune mediators that protect the infant during a critical window of immune immaturity. Secretory immunoglobulin A (sIgA) regulates host–microbe interactions at mucosal surfaces, promoting microbial tolerance while preventing pathogen adherence and invasion. Lactoferrin further supports host defense through its antimicrobial, anti-inflammatory, and immunoregulatory activities, whereas antimicrobial peptides, including defensins and cathelicidins, contribute to pathogen control and epithelial protection. In parallel, microbial-derived metabolites, such as short-chain fatty acids, influence intestinal barrier function and immune cell differentiation, reinforcing the crosstalk between microbial and host compartments (35–38).

Beyond microorganisms and soluble immune mediators, human milk also contains cell-derived extracellular vesicles (EVs), an emerging mechanism of mother-to-infant biological communication. They carry proteins, lipids, messenger RNAs, microRNAs, and other bioactive cargos that can remain biologically active after ingestion. By delivering regulatory signals to intestinal and immune cells, milk-derived EVs are thought to contribute to tissue development, immune maturation, and metabolic adaptation (39).

Collectively, these bioactive components coordinate microbial colonization, immune tolerance, inflammatory regulation, and epithelial development, actively shaping the infant's physiological trajectories. Breastfeeding therefore represents a dynamic biological interface through which maternal microbial, immune, and metabolic signals are continuously conveyed to the infant, contributing to long-term oral and systemic health.

However, the ecological effects of breastfeeding on oral health are influenced not only by milk composition but also by feeding duration, frequency, and timing relative to tooth eruption. Epidemiological studies have suggested that the relationship between breastfeeding duration and ECC is complex and may be influenced by feeding patterns and other coexisting risk factors (40–42). Some studies have reported a higher prevalence of ECC with very prolonged or frequent breastfeeding, particularly when nocturnal feeding continues during the period after tooth eruption, although findings remain heterogeneous across studies (40–42). Evidence regarding breastfeeding during the second year of life remains inconsistent, whereas breastfeeding extending beyond 24 months has been more consistently associated with increased ECC prevalence in observational studies (40, 43, 44). These observations do not indicate that prolonged breastfeeding *per se* is detrimental to oral health, but rather suggest that, after tooth eruption, feeding frequency, nocturnal exposure, oral hygiene, dietary context, and oral clearance capacity may collectively influence caries susceptibility. From a developmental ecological perspective, therefore, the biological benefits of breastfeeding should be considered together with the modulation of feeding patterns and the broader oral environment, to preserve the beneficial host-microbe effects of human milk while minimizing ecological pressures that may favour cariogenic dysbiosis.

### 3.3 Development of the infant oral ecosystem and oral-gut integration

The maturation of the oral microbiota is closely linked to environmental factors, feeding practices, and oral–gut integration, reflecting the continuous interaction between the oral cavity and the gastrointestinal tract (45).

The oral cavity is colonized immediately after birth by pioneer microorganisms, mainly belonging to the genera *Streptococcus*, *Staphylococcus*, *Veillonella*, *Gemella*, and *Rothia*. These pioneer colonizers establish ecological conditions that allow the sequential recruitment of more complex microbial communities. As ecological succession progresses, microbial diversity and community stability increase, particularly following tooth eruption, when new ecological niches become available (46). Longitudinal studies demonstrate that oral and gut microbial maturation occur in parallel, reflecting shared ecological processes during early infancy (47). The composition of these pioneer communities is strongly influenced by perinatal factors. Mode of delivery affects the initial microbial inoculum, with vaginal birth promoting colonization by maternal vaginal and intestinal microorganisms, whereas cesarean delivery is associated with greater exposure to skin- and environment-derived microbes (47).

Feeding practices, together with delivery mode, represent major drivers of microbial succession during the first months of life. Breastfeeding promotes the growth of beneficial microorganisms through the transfer of bioactive compounds, antibodies, and human milk oligosaccharides. In contrast, formula feeding may result in different microbial profiles (48). Subsequent ecological maturation is further modulated by complementary feeding, oral hygiene, family microbial transmission, socioeconomic conditions, and early-life antibiotic exposure, all of which contribute to shaping microbial diversity and ecosystem stability (48). Tongue and oral mucosal hygiene may represent additional, yet relatively underexplored, components of oral ecosystem development during early life. The tongue constitutes an important microbial reservoir, and its microbiota undergoes substantial compositional changes during infancy, with marked shifts occurring during the first year of life and progressive maturation toward an adult-like community by approximately 2 years of age (49). More recent longitudinal evidence indicates that the foundation of the infant tongue microbiota is established by 18 months and that its microbial profile is influenced by early-life factors, including weaning and dietary habits (50). Together with other oral mucosal surfaces, the tongue therefore represents an important ecological habitat that may contribute to the establishment and maintenance of the developing oral microbial ecosystem. Appropriate oral hygiene practices, including tongue hygiene, may contribute to maintaining microbial balance, although evidence specifically addressing their effects on the developing oral microbiota during the first years of life remains limited (51).

Together, these observations reinforce the concept that oral microbiome assembly is a family-driven ecological process rather than the result of maternal transmission alone (47).

As microbial communities mature, ecological interactions extend beyond the oral cavity, reinforcing functional connections with other body ecosystems. This concept is further supported by the oral-gut microbiome axis. The oral cavity and gastrointestinal tract are connected through the continuous swallowing of saliva and oral microorganisms. Although gastrointestinal barriers limit colonization, some oral microbes can survive transit and influence gut microbial communities, particularly under conditions of dysbiosis (45). This oral-gut connection may also represent a mechanistic pathway linking maternal oral dysbiosis and early-life microbial exposure to mucosal immune development. Experimental studies have shown that oral pathobionts can reach the gastrointestinal tract following ingestion and interact with intestinal microbial and immune communities (28). In a maternal periodontitis model, Haraguchi et al. (27) demonstrated that oral pathobionts, including *Klebsiella aerogenes*, were transmitted from mothers to offspring, reached the offspring gut, and altered intestinal immune responses. Notably, increased susceptibility to enteritis persisted into adulthood after the transmitted pathobionts had been cleared, suggesting that transient early-life microbial exposure may induce durable changes in host susceptibility.

Current evidence suggests that oral and gut microbiota communicate through microbial migration and metabolites such as short-chain fatty acids and bile acids. These interactions contribute to immune education, inflammatory regulation, and metabolic homeostasis (52). Taken together, these findings provide a potential mechanistic link between maternal oral dysbiosis, early-life oral microbial exposure, mucosal immune programming, and later disease susceptibility, consistent with the developmental programming perspective of DOHaD (27, 28).

Consequently, oral microbial maturation should be viewed as part of a broader ecological continuum rather than an isolated developmental process. Disruption of this ecological trajectory during early life may increase susceptibility not only to Early Childhood Caries but also to gastrointestinal, immune-mediated, and metabolic disorders (40, 43). These observations support a unified oral-gut ecosystem as a key determinant of health during the first 1,000 days of life.

### 3.4 Tooth eruption as an ecological turning point

Tooth eruption represents an ecological turning point in the infant oral ecosystem. Before eruption, the oral cavity is dominated by desquamating mucosal surfaces, where epithelial turnover and mechanical clearance limit microbial retention. The eruption of teeth introduces hard, non-shedding surfaces that support bacterial adhesion, plaque maturation, and the establishment of new ecological niches (11, 53). This ecological habitat shift creates entirely new microbial niches, driving biofilm maturation, microbial succession, and the first ecological selection pressures within the developing oral ecosystem. Under frequent exposure to fermentable carbohydrates, repeated acidification may drive the transition from ecological homeostasis toward cariogenic dysbiosis (11, 54, 55). Tooth eruption, therefore, represents a critical ecological transition during which microbial communities may diverge toward either health or disease. Although the classical “window of infectivity” for *mutans streptococci* was proposed at 19–31 months, longitudinal studies suggest that cariogenic trajectories may begin much earlier during infancy. Rather than representing a discrete acquisition event, cariogenic colonization reflects an ecological process shaped by microbial succession together with diet, saliva, caregiver-derived transmission, feeding practices, and oral hygiene (56–58). Within the developmental ecological framework proposed in this review, tooth eruption marks the transition from microbial ecosystem establishment to ecological selection, representing one of the most critical windows for the emergence of Early Childhood Caries.

## 4 Dietary ecology of early life

### 4.1 Complementary feeding and oral ecosystem transition

Complementary feeding represents one of the most profound ecological transitions within the developing oral ecosystem. Around 6 months of age, semi-solid and solid foods are gradually introduced alongside breast milk or formula in accordance with World Health Organization (WHO) recommendations (WHO, 2023). This transition modifies not only nutrient intake but also the diversity, frequency, texture, and oral retention of dietary substrates. Compared with a milk-based diet, complementary foods expose the oral cavity to a broader range of fermentable carbohydrates, fibers, proteins, lipids, micronutrients, and food-associated microorganisms (59, 60). These substrates interact with saliva, mucosal surfaces, erupting teeth, and developing biofilms, influencing microbial metabolism and local ecological conditions (11, 54).

The increased availability of dietary substrates promotes microbial succession, favoring increasingly diverse and functionally complex biofilm communities (11, 54). Early oral microbiota, initially dominated by pioneer colonizers such as *Streptococcus* spp., progressively diversify as feeding practices, salivary maturation, tooth eruption, and family microbial transmission generate new ecological niches (14, 61, 62). Consequently, complementary feeding promotes the transition from a relatively simple milk-adapted biofilm toward a more diverse, resilient, and metabolically interactive microbial ecosystem.

Beyond increasing substrate availability, the quality of complementary foods further shapes this ecological trajectory. Diets rich in minimally processed, fiber-containing foods may promote microbial diversity and resilience, whereas frequent consumption of free sugars and refined carbohydrates favors acidogenic and aciduric communities, repeated pH reduction, and cariogenic dysbiosis (11, 54, 63). Together with feeding behaviors, family microbial transmission, antibiotic exposure, and environmental influences, these dietary factors determine whether the developing oral ecosystem progresses toward ecological homeostasis or dysbiosis (63). Complementary feeding should therefore be viewed not simply as a nutritional milestone, but as the first major ecological driver directing oral ecosystem maturation.

### 4.2 Dietary drivers of ecological selection within the oral biofilm

Following the ecological transition associated with complementary feeding, diet becomes the principal environmental force driving ecological selection within the developing oral biofilm. It shapes both the composition and functional activity of the oral microbiome, influencing whether the ecosystem maintains homeostasis or progresses toward cariogenic dysbiosis. According to the Ecological Plaque Hypothesis, dental caries results from diet-driven microbial dysbiosis rather than infection by a single pathogen (64, 65). Long-term dietary habits, therefore, act as persistent ecological pressures that shape microbial community structure and function.

Fermentable carbohydrates are the principal dietary drivers of oral biofilm selection. Frequent exposure to fermentable carbohydrates, rather than absolute sugar intake, generates repeated acidification, selecting acidogenic and aciduric microbial communities. Sucrose is particularly cariogenic because it not only fuels acid production but also promotes extracellular glucan synthesis, enhancing biofilm adhesion and stability (66). Repeated acidification further suppresses health-associated taxa, reinforcing ecological dysbiosis (67, 68).

Free sugars, including those added during food processing or naturally present in honey, syrups, and fruit juices, are readily available for bacterial fermentation and are consistently associated with increased caries risk, supporting current WHO recommendations to limit their intake (69). However, the ecological impact of diet extends beyond sugar quantity alone and is also influenced by food processing, structural characteristics, and overall dietary patterns.

Ultra-processed foods further promote ecological imbalance through their high content of refined carbohydrates and free sugars, together with physical properties that prolong oral retention and facilitate bacterial fermentation (70). The food matrix influences carbohydrate bioavailability, oral clearance, and microbial metabolism, thereby modifying the cariogenic potential of foods (71). In contrast, intact plant foods slow carbohydrate release, stimulate salivary flow, and enhance buffering capacity, whereas highly processed foods increase carbohydrate availability and microbial fermentation.

Diets characterized by high dietary biodiversity and minimally processed plant foods provide fiber, polyphenols, vitamins, minerals, and nitrate-rich compounds that promote microbial resilience, ecological stability, and resistance to cariogenic dysbiosis. Sustainable dietary patterns, including the Mediterranean diet and other predominantly plant-based models, may therefore provide benefits for both oral and planetary health (72).

Overall, diet acts as a continuous ecological regulator of microbial metabolism, biofilm organization, and ecosystem resilience. Accordingly, effective caries prevention should extend beyond sugar restriction toward ecological nutritional strategies based on minimally processed and biodiverse dietary patterns.

### 4.3 Temporal patterns of food intake and acidogenic exposure

The ecological impact of diet on the oral biofilm depends not only on food composition but also on the temporal pattern of dietary exposures. Temporal fluctuations in pH and nutrient availability create ecological pressures that shape microbial selection and biofilm metabolism (11).

Following the ingestion of fermentable carbohydrates, bacterial metabolism generates organic acids that cause a rapid decrease in plaque pH. This phenomenon, originally described by Stephan (1940) (73), is followed by a recovery phase during which salivary buffering and oral clearance gradually restore environmental conditions toward neutrality. The Stephan curve illustrates that the rate and completeness of pH recovery determine both enamel remineralization and biofilm stability (54).

When food intake occurs repeatedly throughout the day, the interval between acidogenic episodes may become insufficient for complete ecological recovery. Consequently, prolonged periods at low pH increase ecological selection for acidogenic and aciduric microorganisms, progressively reducing ecosystem resilience (7, 11).

The distribution of food intake across the 24-hour cycle may further influence these dynamics. Night-time feeding is of particular ecological relevance because salivary flow and buffering capacity are physiologically reduced during sleep, resulting in slower substrate clearance and delayed pH recovery (74, 75). During infancy, however, nocturnal breastfeeding should not be interpreted solely as a cariogenic exposure, as human milk also delivers immune mediators, HMOs, and microbial signals that contribute to host-microbe interactions and ecosystem homeostasis (17, 67). Nevertheless, after tooth eruption, frequent or nocturnal breastfeeding may contribute to caries susceptibility when repeated exposure occurs during periods of reduced salivary flow and delayed pH recovery, particularly in the presence of other risk factors (40–42).

From a developmental ecological perspective, caries results from the cumulative acidogenic load generated by the interaction between exposure frequency, timing, and recovery capacity. Repeated acidification, therefore acts as a chronic ecological stressor, progressively driving the oral ecosystem toward a stable cariogenic biofilm (8).

### 4.4 Meal architecture and oral ecological impact

Traditionally, the relationship between diet and dental caries has been interpreted primarily in terms of nutrient composition and frequency of sugar exposure (76, 77). However, from an ecological perspective, a meal represents a dynamic event that modifies substrate availability, salivary activity, plaque pH, and microbial metabolism, thereby influencing biofilm homeostasis and resilience (8, 11, 78). Building on these established ecological principles, we propose the concept of *Bio-Constructive Meal Architecture* as an author-derived, hypothesis-generating construct, whereby the ecological impact of a meal depends not only on what is eaten but also on how foods are structured, combined, sequenced, and consumed. Food matrix, degree of processing, texture, adhesiveness, and oral retention, influence substrate accessibility and persistence within the oral environment, thereby shaping microbial metabolism and ecological responses (79–81). Foods requiring substantial mastication stimulate salivary flow and oral clearance (74), whereas foods with similar nutrient composition may exert different biological effects according to their physical structure and matrix characteristics (79, 80).

**Figure 1:**
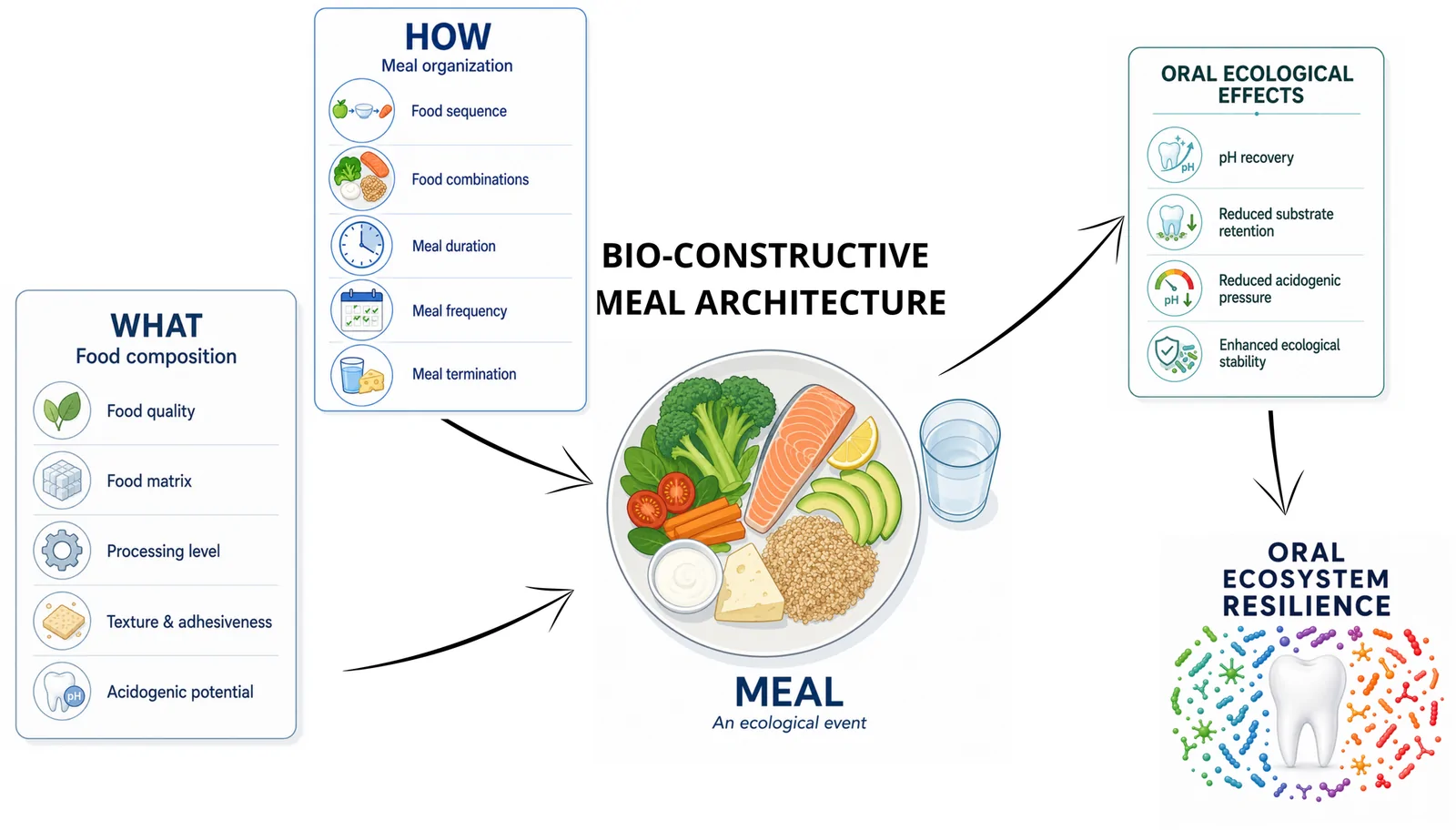
Bio-constructive meal architecture: a conceptual framework linking meal structure to oral ecosystem resilience. The ecological impact of a meal depends not only on food composition (*what is eaten*) but also on meal organization (*how foods are structured within the eating episode*). Food characteristics, including food quality, matrix, processing level, texture, adhesiveness, and acidogenic potential, interact with meal-related factors such as food sequence, food combinations, meal duration, meal frequency, and meal termination strategies. Together, these dimensions shape the meal as a structured ecological event that influences pH recovery, substrate retention, acidogenic pressure, and ecological stability. Through these mechanisms, Bio-Constructive Meal Architecture may promote resilience of the oral ecosystem, facilitate ecological recovery, and support long-term oral health.

As the meal progresses, interactions among foods become increasingly relevant. Food sequence and interactions among different food matrices influence plaque pH dynamics, microbial metabolism, and ecological recovery (11, 54). Acidic foods and beverages may transiently lower oral pH, whereas buffering foods, particularly dairy products rich in calcium, phosphate, and casein, may facilitate pH recovery and restoration of ecological balance (Figure 1) (79, 82). For example, hard cheese has been associated with increased plaque pH and reduced cariogenic pressure through buffering effects and the release of bioavailable calcium and phosphate (83). In addition, minimally processed plant foods rich in dietary fibre may promote mastication and salivary stimulation and may contribute to favourable ecological conditions within the oral environment (84). These observations suggest that the ecological impact of a meal depends not only on individual foods but also on how foods are organized within the eating episode.

The final phase of the meal is characterized by oral clearance and ecological recovery. Salivary buffering and food-induced stimulation of oral clearance mechanisms contribute to the restoration of environmental homeostasis. The capacity of the oral microbiome to recover following dietary perturbations is increasingly recognized as a key component of ecosystem resilience and long-term oral health (8). Taken together, these observations support a conceptual shift toward what may be described as a Bio-Constructive Meal Architecture, in which food selection, food matrix, food sequence, buffering interactions, and meal termination strategies are intentionally organized to promote oral ecosystem resilience, facilitate ecological recovery, and minimize prolonged dysbiotic pressures. Within this framework, a meal is viewed not simply as a source of nutrients but as a structured ecological intervention capable of directing the developmental trajectory of the oral microbiome (Figure 1).

As a proposed construct rather than an established clinical model, *Bio-Constructive Meal Architecture* generates testable predictions. The framework would be supported if meals matched for overall nutrient composition but differing in food matrix, sequence, duration, or termination strategies produced reproducible differences in plaque pH recovery, substrate retention, acidogenic activity, microbial community stability, or other measures of oral ecosystem resilience. Conversely, the absence of such differences in controlled or longitudinal studies would challenge the proposed framework.

## 5 Ecological transition toward a cariogenic biofilm

### 5.1 The cariogenic biofilm as the ecological endpoint of dysbiosis

The oral cavity harbors one of the most complex microbial ecosystems in the human body (13, 85). Within the developmental ecological framework proposed in this review, the cariogenic biofilm represents the ecological endpoint of a progressive transition from a resilient microbial ecosystem toward functional dysbiosis and sustained acidogenic activity (8, 12, 78, 86). Under conditions of frequent fermentable carbohydrate exposure, the oral microbiome undergoes profound shifts that favor the selection and enrichment of acidogenic and aciduric bacterial species. Among these, *Streptococcus mutans* and *Streptococcus sobrinus* are considered primary cariogenic pathogens, owing to their remarkable capacity to metabolize sugars rapidly, producing organic acids, predominantly lactic acid, that drive enamel demineralization (12, 13, 86). *Lactobacillus* species, including *L. acidophilus* and *L. fermentum*, further contribute to caries progression, particularly in dentinal lesions, due to their pronounced aciduric properties, allowing survival and metabolic activity at pH values as low as 4.0–4.5 (65).

Repeated acidic challenges suppress health-associated commensals, including *Streptococcus sanguinis* and *Streptococcus gordonii*, thereby reinforcing ecological dysbiosis (87). Collectively, these ecological changes stabilize the biofilm in a persistent dysbiotic state characterized by sustained acidogenic metabolism and continued enamel demineralization (8, 12, 79).

Central to the structural organization and pathogenic potential of the cariogenic biofilm is the extracellular polysaccharide matrix (EPS), primarily synthesized by *S. mutans* through the action of glucosyltransferases, which convert dietary sucrose into insoluble glucans, principally mutans and dextrans (88). The EPS matrix promotes bacterial adhesion, intercellular cohesion, and the establishment of pH, oxygen, and nutrient gradients that generate metabolically distinct ecological niches while limiting antimicrobial diffusion (89). Biofilm maturation evolves through distinct sequential stages, beginning with reversible attachment of pioneer colonizers to the salivary pellicle, followed by irreversible adhesion, clonal expansion, and the recruitment of secondary colonizers through coaggregation mechanisms (90). Cross-kingdom interactions with *Candida albicans* further enhance EPS production and biofilm virulence (91). As the biofilm matures, three-dimensional tower-like and mushroom-shaped microcolonies emerge, interspersed by water channels that facilitate nutrient transport and metabolic waste removal, while inner anaerobic zones become increasingly dominated by obligate anaerobes and highly aciduric species (90). This architectural complexity renders the mature cariogenic biofilm exceptionally resilient to host defenses and therapeutic interventions.

Rather than representing a simple pathogenic structure, the mature cariogenic biofilm reflects the stable ecological endpoint of progressive ecosystem maladaptation. This perspective supports preventive strategies aimed at preserving microbial resilience during early developmental windows rather than exclusively targeting individual cariogenic microorganisms after dysbiosis has become established (82, 85).

### 5.2 Acidogenic pressure and cariogenic biofilm stabilization

Following the ecological changes described above, persistent acidogenic challenges drive the transition from transient ecological imbalance to a stable cariogenic biofilm (7, 8). Frequent exposure to fermentable carbohydrates imposes a sustained acidogenic load that represents the principal ecological driver of cariogenic dysbiosis. When dietary sugars are metabolized by acidogenic members of the biofilm community, particularly *Streptococcus mutans* and *Lactobacillus* species, organic acids, predominantly lactic acid, are rapidly generated, causing a sharp decline in plaque pH (92). As illustrated by the Stephan curve, plaque pH rapidly falls below the critical threshold for enamel demineralization before gradually recovering through salivary buffering and microbial metabolic neutralization (11). The efficiency of this recovery phase is a major determinant of ecosystem resilience (7, 8). When sugar exposure occurs repeatedly throughout the day, the intervals between acid challenges become insufficient to allow complete pH recovery. The biofilm microenvironment consequently remains in a state of chronic acidification, and this sustained low-pH condition constitutes a powerful selective ecological pressure (78, 92). Acid-sensitive commensal species, such as *Streptococcus sanguinis*, are progressively displaced, whereas acidogenic and aciduric organisms gain a competitive advantage and dominate the biofilm (65, 78). The resulting pH dysregulation establishes a self-reinforcing pathological cycle: as acidogenic bacteria become increasingly dominant, their metabolic output further acidifies the biofilm environment, amplifying mineral dissolution and deepening the ecological disruption (65, 92). Overall, this cascade, from repeated acidogenic load to selective ecological pressure, progressive pH dysregulation, and impaired recovery capacity, illustrates how repeated acidogenic perturbations progressively reshape the oral ecosystem, ultimately driving toward a dysbiotic, cariogenic biofilm. These observations further support preventive strategies aimed at preserving ecosystem resilience by targeting the ecological determinants of dysbiosis, including dietary drives, rather than focusing exclusively on the elimination of individual microbial species (7, 8).

## 6 Strategies for oral ecosystem modulation

### 6.1 Maternal-centered ecosystem

Growing evidence suggests that maternal oral dysbiosis may influence pregnancy outcomes through systemic inflammatory and microbial pathways. Recent experimental evidence further suggests that maternal periodontal inflammation may extend beyond the oral cavity, affecting the maternal gut microbiome and intestinal inflammatory responses (20). Pathological hematogenous translocation of specific oral pathobionts has been reported in selected adverse pregnancy outcomes, including their detection in placental or fetal tissues (85, 86). The report of term stillbirth associated with *Fusobacterium nucleatum* provides an illustrative example of pathological microbial translocation (85). However, this evidence should not be interpreted as demonstrating the existence of a resident placental or amniotic microbiota in healthy pregnancy. Indeed, the presence of a physiologically resident microbiota in the placenta and amniotic environment remains controversial (93). Microbial signals detected in fetal tissues, placenta, and amniotic fluid may instead be attributable to contamination during sampling, DNA extraction, or sequencing, particularly in these low-biomass environments. Similarly, Panzer et al. (94) in a re-analysis of 15 publicly available placental microbiota datasets, found that previously reported microbial signals were strongly influenced by environmental contamination and mode of delivery and did not provide consistent evidence for a resident placental microbiota. Banchi et al. (95) likewise concluded that current evidence in humans and animals supports, at most, the presence of low-level bacterial signals in fetomaternal tissues, while the viability and biological significance of such signals remain uncertain. Accordingly, the prenatal component of the developmental-ecological framework proposed here does not require the presence of a resident fetal or placental microbiota, but may instead be mediated by maternal inflammatory, immune, metabolic, and microbial signals that shape the fetal developmental environment.

The oral microbiome begins its development long before tooth eruption and is profoundly influenced by maternal microbial reservoirs and broader maternal microbial ecosystems (96). According to the Developmental Origins of Health and Disease (DOHaD) paradigm, microbial, inflammatory, nutritional, and metabolic exposures occurring during the maternal and early-life periods may influence long-term health trajectories, including oral health outcomes (97). Maternal oral health represents a major determinant of microbial transmission to the infant. Mothers affected by untreated dental caries or periodontal disease harbor increased levels of cariogenic and inflammatory microorganisms, including *Streptococcus mutans* and other oral pathobionts, which may be vertically transmitted through salivary contact and shared caregiving practices (98). Importantly, maternal oral dysbiosis may therefore influence the infant developmental environment through both direct microbial transmission and broader inflammatory and metabolic effects. The potential consequences of maternal oral dysbiosis for the infant gut and mucosal immune development are further discussed in the context of the oral-gut axis (Section 3.3). Evidence suggests that maternal microbial profiles are predictive of the early oral colonization patterns observed in children (14).

Consequently, maternal-centered ecosystem modulation should begin before conception and continue throughout pregnancy. Professional oral care, periodontal management, healthy dietary habits, microbial diversity preservation, and prudent antibiotic use represent key strategies for promoting a healthy oral ecosystem in the child. Such interventions may reduce dysbiotic microbial transmission and future risk of dental caries and oral inflammatory diseases (97).

Emerging microbiome-based approaches include probiotic supplementation. Oral strains such as *Streptococcus salivarius* K12 and M18 have been investigated for their potential to modulate oral microbial communities through bacteriocin production and ecological competition (99, 100). In addition, maternal supplementation with *Limosilactobacillus reuteri*, *Lacticaseibacillus rhamnosus* GG, and *Bifidobacterium animalis* subsp. *lactis* BB-12 may support microbial homeostasis and immune maturation during pregnancy, although their effects on maternal–infant oral microbial transmission remain to be clarified (101). Overall, probiotic supplementation may represent a promising adjunctive strategy during pregnancy; however, robust evidence demonstrating a direct effect on maternal–infant oral microbial transmission is still lacking.

### 6.2 Infant-centered ecosystem modulation

The neonatal oral microbiome begins to develop immediately after birth and undergoes rapid ecological succession during the first months of life. Delivery mode, feeding practices, maternal microbial exposure, and environmental factors collectively shape the long-term organization of the oral ecosystem (14). Continuous maternal-infant microbial exchange occurring during breastfeeding further contributes to oral microbiome maturation.

Complementary feeding represents a second critical developmental window. Introduction of diverse minimally processed foods promotes microbial richness and ecological resilience, whereas early and frequent exposure to free sugars favors ecological dysbiosis and increases the risk of dental caries (102).

Limiting the frequency of sugar exposure may help preserve oral ecosystem stability and reduce selective pressures favoring cariogenic biofilm development (103).

Establishing a diverse and resilient oral ecosystem during infancy may represent a key strategy for reducing susceptibility to later dysbiosis and caries.

Recent studies have also explored the role of oral probiotics in supporting microbial homeostasis during early life. Supplementation with oral commensals such as *Streptococcus salivarius* K12 and M18 may support microbial homeostasis through ecological competition (99, 100). Further studies are ongoing on other strains or bacteria as promising oral cavity probiotic candidates, like *Str. salivarius* ST48HK, ST59HK, ST61HK, and ST62HK; *Lb. plantarum* ST63HK and ST66HK; *Lb. sakei* ST69HK; and *Lb. gasseri* ST16HK (104).

Beyond currently available probiotics, increasing attention is being directed toward so-called next-generation oral probiotics. Among next-generation oral probiotics, *Streptococcus dentisani* and *Weissella cibaria* have shown potential to enhance ecological stability, modulate oral biofilm composition, and promote colonization resistance against opportunistic pathogens (105, 106).

Recent evidence suggests that the neonatal oral microbiome may represent an early biomarker of future cariogenic susceptibility. Rather than focusing exclusively on the acquisition of specific pathogens, current research supports the concept that alterations in microbial succession and ecosystem maturation during the first thousand days of life may predispose children to the later development of a cariogenic oral environment (16, 107).

### 6.3 Biofilm- and microbiota-targeted strategies

The oral cavity harbours one of the most diverse microbial ecosystems in the human body, comprising over 700 bacterial species organised in structured biofilms that interact dynamically with the host (108). Accordingly, oral ecosystem modulation represents a paradigm shift from non-selective antimicrobial approaches toward precision strategies that preserve microbial diversity while selectively reducing pathobionts (65). Beyond diet and salivary function as a gatekeeper of oral microbial balance, fluoride remains a cornerstone of caries prevention, with some authors describing dental caries as a “fluoride deficiency disease” (109, 110). It acts through enamel remineralisation and by inhibiting bacterial enolase and glycolytic metabolism, thereby reducing biofilm acidogenicity and limiting the ecological advantage of acid-tolerant species (111). Xylitol, a non-fermentable sugar, modulates biofilm composition by limiting substrate availability for acidogenic bacteria and interfering with *S. mutans* adhesion and persistence within the biofilm (112). Arginine, metabolised via the arginine deiminase system by health-associated commensal species such as Streptococcus parasanguinis, elevates plaque pH and promotes the competitive dominance of health-associated alkali-generating bacteria over aciduric pathobionts, effectively reshaping the biofilm's biochemical environment toward a less dysbiotic state (113). Probiotic supplementation introduces exogenous beneficial microorganisms capable of colonising oral surfaces, producing bacteriocins, and competitively excluding cariogenic and periodontopathic species through direct bacterial interference (114). Complementing this approach, postbiotics, defined as preparations of inanimate microorganisms and their bioactive components, offer a potentially safer and shelf-stable alternative, delivering metabolites such as short-chain fatty acids, exopolysaccharides, and antimicrobial peptides (115). Despite encouraging results, the standardization of strains, dosage regimens, and assessment of long-term clinical outcomes remains an area of ongoing investigation. Other emerging ecological approaches are based on the concept of bacterial interference, whereby natural microbial competition is exploited to promote colonisation resistance against pathogenic species. Bacterial interference strategies further exploit natural microbial competition by deliberately introducing low-virulence strains to occupy ecological niches and prevent colonisation by more pathogenic counterparts, as demonstrated by genetically attenuated S. mutans strains designed to displace their wild-type equivalents within established biofilms (116). Collectively, these approaches underpin microbiome-based prevention, a framework grounded in Ecological Plaque Hypothesis principles that aims to maintain symbiotic microbial communities rather than achieving sterility (11). Furthermore, advances in metagenomics and salivary microbiome profiling now enable microbiome-informed risk stratification, allowing clinicians to identify dysbiotic signatures predictive of caries or periodontitis onset before clinical manifestation (117). Together, these advances may facilitate the development of personalised, ecology-driven preventive protocols informed by microbial community dynamics and individual risk profiles (13).

## 7 A one health ecological model of early-life caries development

### 7.1 Developmental ecology framework and clinical implications

The evidence reviewed throughout this manuscript supports a shift from a pathogen-centered interpretation of early childhood caries (ECC) toward a developmental and ecological model in which oral health emerges from dynamic interactions among microbial communities, dietary exposures, host factors, behaviors, and environmental influences (11, 66, 78, 86). Within this framework, ECC is best understood as the result of progressive ecological dysbiosis affecting the developing oral ecosystem rather than the action of specific pathogens alone (8, 14, 78).

This perspective is consistent with the One Health paradigm and the Developmental Origins of Health and Disease (DOHaD) framework, which recognize that biological and environmental exposures interact throughout early life to shape future health trajectories and disease susceptibility (118, 119). The trajectory toward oral health or disease may be influenced during prenatal life, long before clinical caries appears, through maternal microbial, nutritional, inflammatory, immune, metabolic, and environmental influences (120). These prenatal influences may act through maternal inflammatory and metabolic signaling, immune modulation, and effects on fetal developmental processes, including enamel formation (120, 121).

**Figure 2:**
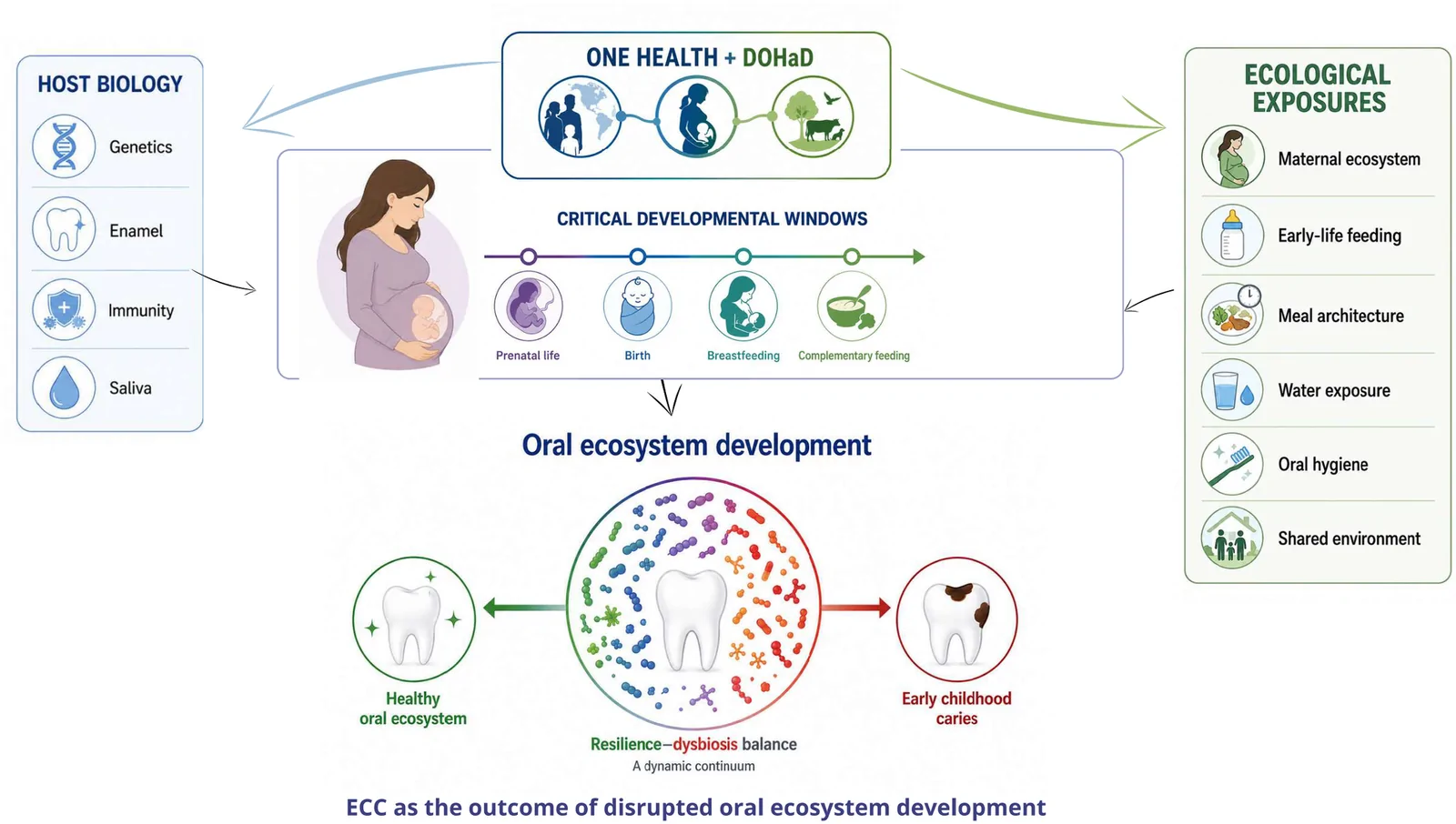
Developmental ecology of the infant oral ecosystem: a conceptual framework. The framework illustrates how host biological factors and ecological exposures interact across critical developmental windows to shape oral ecosystem development within the One Health and DOHaD paradigms. The prenatal window reflects the influence of the maternal-fetal developmental microenvironment on subsequent oral ecosystem trajectories. The balance between ecological resilience and dysbiosis ultimately determines whether the developing oral ecosystem follows a trajectory toward health or Early Childhood Caries (ECC).

Developmental enamel defects, including enamel hypoplasia and hypomineralisation, represent an important host-mediated pathway through which prenatal and perinatal exposures may influence subsequent caries susceptibility. A systematic review and meta-analysis found an association between developmental enamel defects and ECC, with a particularly strong association for enamel hypoplasia, although the certainty of the evidence was limited (122). Preterm birth and low birth weight, particularly very low birth weight, have also been associated with developmental enamel defects in the primary dentition and with enamel hypoplasia (123). Maternal prenatal vitamin D status may additionally influence enamel development, although the available evidence remains heterogeneous (124). Hypomineralised second primary molars (HSPM) represent another clinically relevant developmental enamel defect and may identify children requiring closer preventive surveillance, although evidence linking HSPM specifically to caries in the primary dentition remains limited (125). Thus, structurally or mineralically compromised enamel may increase susceptibility to acid-mediated demineralisation under cariogenic ecological conditions, illustrating how prenatal and perinatal exposures can shape later ECC vulnerability through host biology rather than through direct fetal microbial colonization (Figure 2).

Importantly, this prenatal component does not require the presence of a resident placental or amniotic microbiota, but may instead reflect the broader maternal developmental environment, as discussed above.

From an ecological perspective, the maternal ecosystem constitutes the child's first biological environment and may represent the earliest opportunity for oral health promotion. Maternal oral health, microbial ecology, and shared environmental exposures contribute to the establishment of the infant oral microbiome through vertical transmission and early microbial succession (14, 126, 127). Consequently, maternal health promotion during preconception, pregnancy, and lactation should be regarded as an integral component of caries prevention.

Following birth, breastfeeding and complementary feeding represent critical ecological windows during which microbial, immunological, and nutritional factors contribute to oral ecosystem development (14, 119, 128, 129). Beyond nutrient composition alone, dietary diversity, early sensory experiences, and exposure to minimally processed foods may contribute to healthier dietary trajectories and potentially support microbial ecosystem resilience (130). Building on these observations, the concept of Bio-Constructive Meal Architecture proposes that oral health is influenced not only by food selection but also by the ecological organization of eating episodes. Meal structure, food matrix interactions, and oral recovery dynamics may collectively shape microbial resilience and ecosystem stability (8, 11) (Figure 2).

Clinically, these observations support a broader ecological approach to risk assessment that extends beyond traditional dental risk factors and incorporates maternal influences, early-life feeding patterns, developmental history, family behaviors, and environmental exposures (131). Ultimately, ECC may be regarded as a biological marker of disrupted host-microbe-environment interactions occurring during the first thousand days of life (14, 119). Host biological factors, including genetic determinants of enamel development, salivary composition, immune function, and epigenetic programming, may further modulate individual susceptibility to ecological dysbiosis and ECC (9, 10). Prevention can therefore be broadened to include strategies that build and maintain a resilient oral ecosystem through a sequence of favourable ecological exposures operating from maternal life through early childhood. This developmental-ecological perspective is intended to complement, rather than replace, established evidence-based preventive measures, including fluoride use, dietary sugar control, oral hygiene, and regular dental care.

### 7.2 Strengths and limitations

The principal strength of this review lies in the development of an integrated conceptual framework that brings together evidence from oral microbiology, developmental biology, nutritional ecology, microbiome science, and the One Health and DOHaD paradigms (109, 110). By reframing early childhood caries (ECC) as a disorder of the developmental ecology of the infant oral ecosystem, this review provides a broader biological perspective and proposes a conceptual framework that may inform future preventive strategies and research. As a narrative review, this work does not follow a formal systematic review methodology and may therefore be subject to selection bias despite a structured literature search. Furthermore, several components of the proposed framework are currently supported mainly by observational, mechanistic, or preclinical evidence (8, 14, 16), and causal relationships remain to be confirmed through longitudinal birth cohort studies and well-designed interventional research (14, 16, 17). Accordingly, the Developmental Ecology framework should be regarded as a conceptual model that integrates current evidence and generates testable hypotheses rather than as a fully validated causal model.

### 7.3 Future perspectives

The developmental-ecological framework proposed in this review suggests that future preventive strategies for early childhood caries (ECC) should increasingly focus on shaping oral ecosystem development rather than simply controlling established disease. Emerging evidence indicates that oral health trajectories may be influenced by factors operating well before disease onset, creating opportunities for preventive interventions across multiple developmental stages (14, 119) (Table 1). These opportunities extend from the preconception period to early-life nutrition, microbiome-directed therapies, and precision ecological prevention.

**Table 1:** Emerging directions for ecological prevention and translational research in early childhood caries.

Future area	Scientific rationale	Research and translational implications	Key references
Preconception oral ecosystem optimization	Maternal microbial communities contribute to early microbial transmission and may influence oral ecosystem assembly.	Integration of oral health into preconception and prenatal care; investigation of maternal ecological determinants of offspring oral health.	(14, 126, 132)
Ecologically designed infant nutrition	Human milk bioactive components influence microbial colonization, host–microbe interactions, and immune maturation.	Development of microbiome-targeted nutritional strategies, including HMOs and postbiotic-enriched formulations when breastfeeding is not possible.	(35, 133)
Ecological biomarkers and functional risk assessment	Microbial resilience, community interactions, and metabolic functions may better reflect disease susceptibility than individual taxa.	Development of ecological biomarkers and functional microbiome profiling tools for personalized caries risk assessment.	(7, 8, 86)
Functional biofilm modulation	Biofilm ecology may be influenced by targeting microbial functions, communication pathways, and ecological interactions rather than indiscriminate microbial elimination.	Investigation of postbiotics, bacteriocins, quorum-sensing inhibitors, and microbiome-directed therapies for ecological biofilm management.	(134, 135)
Longitudinal ecological research across the first 1,000 days	Oral ecosystem development is a dynamic process shaped by biological, nutritional, behavioral, and environmental exposures.	Prospective birth cohorts integrating microbial, nutritional, behavioral, and environmental data to identify critical developmental windows and validate ecological biomarkers.	(14, 17, 119)
Precision ecological prevention	Integration of developmental biology, oral microbiomics, and ecological risk assessment may support individualized prevention strategies.	Development of personalized prevention programs based on ecological profiles, developmental trajectories, and ecosystem resilience.	(8, 86, 115)

The developmental-ecological framework proposed in this review supports a shift from disease-centered prevention toward strategies aimed at promoting oral ecosystem development, resilience, and long-term ecological stability. The table summarizes the main future research areas and their potential translational applications.

Maternal microbial communities contribute to early microbial transmission, while oral microbial colonization follows a dynamic ecological succession during infancy (14, 126, 132).

Another emerging area concerns ecologically designed infant nutrition. Human milk oligosaccharides (HMOs) and other milk-derived bioactive compounds contribute to microbial and immune development (35, 133).

Advances in oral microbiomics are also shifting attention from pathogen-centered models toward ecological biomarkers of health (8, 86).

Novel opportunities may arise from functional biofilm modulation and ecological engineering of the oral microbiome. Rather than focusing exclusively on microbial eradication, future interventions may aim to promote ecological recovery, resilience, and biofilm homeostasis through postbiotics, bacteriocins, quorum-sensing modulators, microbiome-directed therapies, and engineered microbial approaches (134–137).

Finally, translational research should move beyond cross-sectional descriptions of microbial composition and focus on longitudinal ecological trajectories across the first 1,000 days of life (17, 119). The principal emerging research directions and their potential translational applications are summarized in Table 1.

Ultimately, the integration of developmental biology, microbiome science, and One Health principles may enable a transition from disease-centered prevention toward proactive ecological stewardship of the developing oral ecosystem (118, 138).

## 8 Conceptual conclusions

The evidence synthesized in this review supports a paradigm shift in the understanding of Early Childhood Caries (ECC). Rather than representing a disease initiated after tooth eruption by specific cariogenic microorganisms, ECC may be viewed as the clinical manifestation of disrupted developmental ecology of the infant oral ecosystem, consistent with the ecological plaque hypothesis and the emerging concept of oral ecosystem resilience.

Within this framework, host biology, including genetic susceptibility, enamel development, salivary function, and immune function, interacts continuously with maternal microbial inheritance, early microbial succession, breastfeeding, complementary feeding, dietary ecology, tooth eruption, and environmental exposures to shape microbial resilience throughout the first 1,000 days of life.

This perspective redefines the cariogenic biofilm as the ecological endpoint of cumulative developmental processes rather than the primary initiating factor of disease. Consequently, prevention should be broadened to incorporate ecological strategies that complement established preventive measures, including pathogen control, dietary sugar control, fluoride use, oral hygiene, and regular dental care, while also promoting maternal oral health, breastfeeding, healthy nutrition, family-centered preventive behaviors, and microbial resilience from the earliest stages of life within a One Health framework.

Taken together, the available evidence supports a Developmental Ecology of Early Childhood Caries, proposed in this review as a conceptual framework in which oral health emerges from the dynamic interaction among host biology, microbial ecology, nutrition, behavior, and the environment throughout the first 1,000 days of life. This framework is intended to organize current evidence and generate testable hypotheses for future longitudinal and interventional studies, rather than to represent a fully validated causal model.
